# Network Meta-Analysis of Trials Testing If Home Exercise Programs Informed by Wearables Measuring Activity Improve Peripheral Artery Disease Related Walking Impairment

**DOI:** 10.3390/s22208070

**Published:** 2022-10-21

**Authors:** Shivshankar Thanigaimani, Harry Jin, Munasinghe Tharindu Silva, Jonathan Golledge

**Affiliations:** 1 The Queensland Research Centre for Peripheral Vascular Disease (QRC-PVD), College of Medicine and Dentistry, James Cook University, Townsville, QLD 4811, Australia; 2 The Australian Institute of Tropical Health and Medicine, James Cook University, Townsville, QLD 4811, Australia; 3 The Department of Vascular and Endovascular Surgery, Townsville University Hospital, Townsville, QLD 4814, Australia

**Keywords:** home exercise, wearable, physical activity, walking ability, maximum walking distance, 6-minute walking distance

## Abstract

**Background:** This study aimed to investigate whether home exercise programs informed by wearable activity monitors improved walking ability of patients with peripheral artery disease (PAD). **Methods:** A systematic literature search was performed to identify randomised controlled trials (RCT) testing home exercise that were or were not informed by wearable activity monitors. The primary outcome was the change in walking distance measured by a six-minute walking test or treadmill test over the course of the trial. Network meta-analysis (NMA) was performed using the gemtc R statistical package. The risk of bias was assessed using Cochrane tool for assessing risk of bias in RCTs (RoB 2.0). **Results:** A total of 14 RCTs involving 1544 participants were included. Nine trials used wearable activity monitors to inform the home exercise program tested, while five trials did not use wearable activity monitors to inform the home exercise program tested. Overall quality assessment showed 12 trials to be at low risk of bias and two trials at high risk of bias. Home exercise programs informed by wearable activity monitors significantly improved walking distance compared to non-exercise controls (Mean difference, MD: 32.8 m [95% credible interval, CrI: 6.1, 71.0]) but not compared to home exercise programs not informed by wearable activity monitors (MD: 4.7 m [95% CrI: −38.5, 55.4]). **Conclusions:** Home exercise informed by wearable activity monitors improve walking ability of patients with PAD. It is, however, unclear if activity monitoring informed exercise programs are more effective than exercise programs not using activity monitors.

## 1. Introduction

Exercise therapy is an evidence-based treatment for peripheral artery disease (PAD) recommended in all current practice guidelines which can be delivered by central facility supervised or home programs [1,2,3]. Most exercise programs include walking, which needs to regularly induce moderate to severe leg pain to improve function, which is challenging for PAD patients [4,5]. The uptake of and adherence to supervised exercise programs is limited, possibly due to the inconvenience of regularly attending a central facility [6]. Home exercise is an effective alternative treatment for walking impairment in people with PAD which does not require attendance at a central facility [2,7]. This is usually performed by patients exercising under their own direction with support from the treatment centre [7]. This support can consist of a wide range of options such as written advice, one or more visits to the treatment centre for instruction and training, and various forms of remote support (e.g., telephone coaching) [7]. The lack of direct monitoring and supervision by a health professional has been suggested to limit efficacy of such programs [7].

There are a range of established ways for self and remote monitoring of the intensity and volume of exercise using devices worn by patients which could potentially replace direct supervision and improve efficacy of home exercise programs. This includes accelerometers or pedometers worn to monitor exercise volume and intensity, which have been used to provide data to inform motivational counselling to promote exercise [8,9,10]. Randomised controlled trials (RCT) in healthy populations have suggested that use of such wearables can increase physical activity [11,12]. A number of RCTs in participants with PAD have compared home exercise programs informed by wearable activity monitors with control groups not receiving a structured exercise program [4,13,14,15,16]. Home exercise programs not informed by wearable activity monitors have also been compared with similar control groups [17,18]. However, there have been no RCTs directly comparing home exercise programs informed or not informed by wearable activity monitors in management of patients with PAD. Analysis of data from these RCTs by network meta-analysis (NMA) can enable indirect assessment of the benefit of home exercise programs informed by wearable activity monitors for treatment of PAD. We aimed to perform a NMA to test whether home exercise programs informed by wearable activity monitors improved walking ability of patients with PAD compared to non-exercise controls and home exercise programs not informed by wearable activity monitors. The main outcome was improvement in walking function assessed through either six-minute walking distance (6MWD) or treadmill maximum walking distance (TMWD). We hypothesised that use of home exercise programs informed by wearable activity monitors would significantly improve walking ability in patients with PAD.

## 2. Methods

### 2.1. Search Strategy

This review was performed according to the Preferred Reporting the Items for Systematic Review and Meta-Analysis (PRISMA) statement [19]. The study protocol was registered with PROSPERO (CRD42022308138). The literature search was conducted by two authors (ST, JG). The databases PubMed and Cochrane Central Register for Controlled Trials were searched on 21 April 2022. The search strategy included the synonyms or similar terms of “Wearables” AND “PAD” and a detailed search string is provided in Supplementary Material. References from the included studies were also examined for eligible studies. No date or language restrictions were applied. Inclusion criteria were RCTs testing home exercise programs either informed or not informed by wearable activity monitors and were compared against a usual care non-exercise control group in participants with PAD. This control group was not taking part in a structured exercise program. To be considered as wearables group, studies were required to have used data obtained from a wearable activity monitor to inform the exercise program. This could have been by the participant self-monitoring their physical activity measured by the monitor and/ or by a treatment team monitoring this and using the activity data to inform the support they provided. For inclusion, the RCT needed to report the baseline and follow-up walking performance assessed as 6MWD or TMWD. If multiple studies were published from a clinical trial registered under the same number, all studies were included either when follow-up period were at least 6 months apart, or components of the home exercise intervention varied or different primary outcomes were published separately (e.g., 6MWD or TMWD). Minimum data for study inclusion were walking distance reported at baseline and the end of follow-up or change in walking distance for participants in each group. Exclusion criteria included: non-randomised studies, case studies, letters, studies in which all participants did not have diagnosed PAD and trials that did not compare home exercise programs against a non-exercise control group. Eligibility was assessed by two authors (ST, JG). Any discrepancies were resolved through discussion.

### 2.2. Data Extraction and Outcomes

Data were extracted on a customised spreadsheet by three authors (ST, HJ, MTS). The primary outcome of this study was improvement in walking performance defined as mean change in 6MWD or TMWD at the completion of follow-up. The change in walking distance for participants was calculated using outcomes reported at baseline and completion of follow-up and reported separately in the following groups: Home exercise programs informed by wearable activity monitors, home-exercise programs not informed by wearable activity monitors and non-exercise controls. Home exercise programs informed by wearable activity monitors included any home exercise program which used any kind of sensors or wearables to monitor the physical activity and/or adherence, and use the information to support the home exercise program. Home exercise programs not informed by wearable activity monitors were defined as any home exercise program that did not use any kind of wearables during the follow up period to inform the exercise programs. Controls were defined as participants not taking part in any kind of structured exercise program. In studies that did not report the change score, mean difference (MD) was calculated as the difference in mean values between the specified follow-up and baseline. Standard deviation of change scores was imputed using the formula [20]: sd_change_ = √[sd^2^_baseline_+ sd^2^_Final_ − (2× Corr × sd_baseline_× sd_Final_)]. Pearson correlation coefficient was (Corr) calculated using individual participant data from one trial [16] as used previously [2,7] was employed in the sd_change_ formula. In studies that reported confidence interval, standard error (SE) was calculated using the formula: (upper limit − lower limit)/3.92. The derived SE was used to calculate standard deviation using the formula: SE/√(sample size) [21]. In studies that reported walking time, distance was calculated using the formula: distance = speed × time. Pooled means were calculated using the formula [Pooled mean = (Sample size^1^ + Sample size^n^/Total number of trials]. Pooled SD was calculated using the formula [Average of SD = √((N^1^ − 1) × SD^1^^2 + … (N^n^ − 1) × SD^n^^2)/((N^1^ + … N^n^) − n), where n is the total number of studies [22]. The following information was also extracted from the included trials: Sample sizes for groups; components included in the exercise programs, such as total number of facility visits, session frequency and length, session structure, educational sessions, online sessions, behavioural counselling, follow-up duration, age, sex, body mass index (BMI), ankle brachial pressure index (ABPI), history of diabetes, myocardial infarction, stroke, coronary artery disease and prior lower limb revascularisation at baseline.

### 2.3. Quality Assessment

Three authors (ST, HJ, MTS) independently assessed the risk of bias of all included studies using the Cochrane tool for assessing risk of bias in RCTs (RoB 2.0) [23]. The trials were assessed as either at low risk of bias, some concerns (probably low risk of bias) or high risk of bias based on the following domains: randomisation process, deviation from intended interventions, missing outcome data, measurement of outcomes and selective reporting. We rated trials at high risk of bias overall if one or more domains were rated as ‘some concerns-probably high risk of bias’ or as ‘high risk of bias’, and as low risk of bias overall if all domains were rated as ‘some concerns-probably low risk of bias’ or ‘low risk of bias’. Any inconsistencies were resolved through discussion between the authors until a consensus was reached. No studies were excluded on the basis of risk of bias.

### 2.4. Data Analysis

The Bayesian random-effects NMA was performed using the R statistical package “gemtc” by assuming consistency, variance scaling factor of 2.5, non-informative weakly prior distribution of standard deviation between trials [24]. Markov Chain Monte Carlo (MCMC) simulations were performed to estimate the posterior distributions of change scores between different arms by running the simulations long enough (100,000 iterations) to reach accurate estimates for the model, and the convergence and stability of the network models were assessed using trace and density plots and Potential Scale Reduction Factor (PSRF), respectively. Inconsistency of the network model could not be assessed as there were no head to head trials identified [25]. Ranking probability was calculated for all available treatment strategies for each follow-up time and surface under the cumulative ranking curve (SUCRA) plot was developed [26]. All included studies were considered as independent data for analysis even if registered under the same trial number. Meta-regression based on data availability in a minimum of three trials was performed to test the effect of length of the exercise program (weeks), whether or not telephone counselling was provided (yes/no) and number of facility visits on the walking performance. Estimates were reported as mean difference (MD) and 95% credible interval (CrI) of the walking distance change score. A *p*-value of <0.05 was considered as statistically significant. All statistical analyses were performed using R program version 4.2.1.

## 3. Results

### 3.1. Study Selection

**Figure 1:**
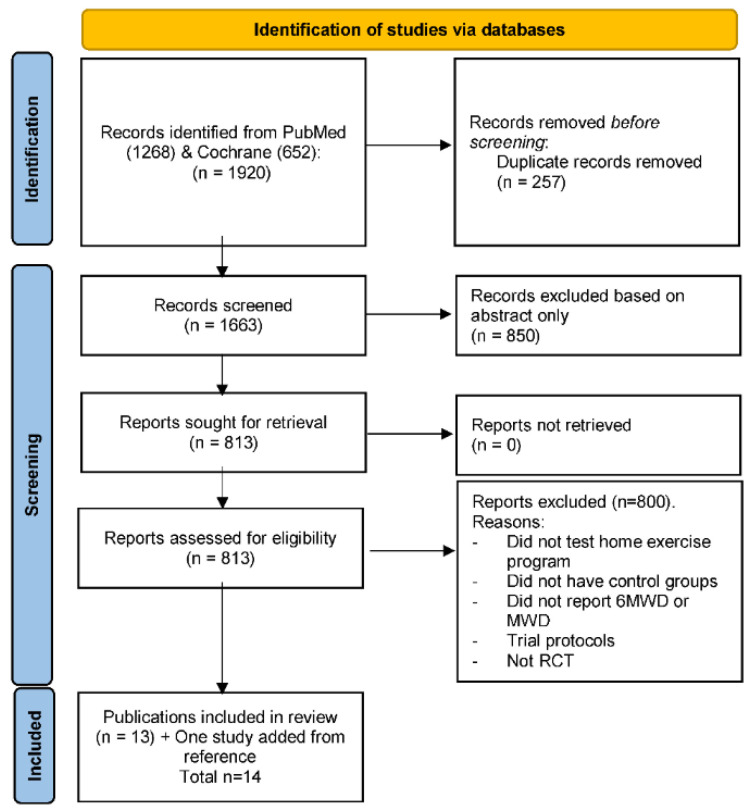
Preferred Reporting Items for Systematic Reviews and Meta-Analyses flow diagram. A total of 1920 publications were identified, 1663 publications were screened and after exclusion of irrelevant studies, 14 publications were included. 6MWD—Six-minute walking distance; MWD—Maximum walking distance; RCT—Randomised controlled trial.

The literature search identified 1920 studies, of which 1663 unique records were assessed (Figure 1). Ultimately, 14 RCTs with 1526 participants were included for analysis [4,13,14,15,16,18,27,28,29,30,31,32,33,34]. Of these, nine RCTs tested home exercise programs informed by wearable activity monitors and reported either 6MWD [4,13,15,16,27] or TMWD [28,29,30,31]. Five trials tested home exercise programs not informed by wearable activity monitors and reported either 6MWD [14,18,32] or TMWD [33,34] were included. One trial included two different types of home exercise programs not informed by wearable activity monitors which were considered as two separate arms for analysis [32]. Two studies from the same trial registration reported 6 month follow-up of exercise intervention [14], and 12 month follow-up of exercise intervention and telephone coaching at 7-12 months [18]. Another two studies from same trial registration reported 6MWD [13] and TMWD [30] published separately.

### 3.2. Patient Characteristics

Where reported, the baseline risk factors were not significantly different between participants allocated to the home exercise programs and control groups (Table 1). Pooled estimates of the prevalence of risk factors in participants allocated to the home exercise programs and control groups are shown in Table 1.

### 3.3. Study Characteristics

The structures of the exercise programs tested in the included RCTs and how activity monitoring data were used to manage the programs are summarised in Table 2 and Table 3. In brief, exercise frequency typically ranged between 3 and 5 times per week, with each session lasting between 15 and 50 min in all of the included trials. In four trials with program duration ranging between 12 and 52 weeks, facility visits took place every week for the first 2–6 weeks followed by telephone counselling for the remainder of the program [4,13,15,27]. Two trials had facility visits every week throughout the program duration of 6 [16] and 24 [14] weeks and one study had facility visits for first 6 months followed by another 6 months of telephone counselling [18]. Ten trials provided telephone counselling to the home exercise group [4,15,16,18,27,28,29,32,33,34]. Eight studies provided behavioural counselling through motivational interviewing to all participants through a trained facilitator [4,14,15,16,18,27,28,32]. Nine trials provided education counselling in person or through telephone as part of the exercise program [4,14,15,16,18,27,28,29,32]. In all the studies that used wearables to inform the home exercise program, investigators used the data for counselling and patients were able to view their data during the study in 8 trials [4,13,15,16,27,29,30,31]. Patients were encouraged to use the data for self-motivation in six studies [4,13,15,16,27,30].

One possible advantage to activity monitoring could be to encourage adherence to the prescribed exercise program. There was, however, limited reporting of this within the included trials (Table 2). Only seven trials reported adherence, of which six trials reported exercise intensity of the participants measured using different methods including a validated 7-item questionnaire [27] and data from activity monitor [4,13,14,15,30]. Attendance rates of participants at site visits were reported to vary between 81 and 93% in three trials [4,15,27]. Attendance rates at scheduled telephone intervention calls were reported to vary between 74% and 85% [4,15,27]. Two trials reported that participants completed 81 and 83% of the allocated exercise sessions [13,30]. Two trials reported that participants performed an average of 3.5 ± 1.5 and 3.5 ± 4.2 exercise sessions per week [4,15]. One trial reported that 84% of the participants attended the scheduled group-mediated cognitive behavioural intervention [14]. One trial used a six-item questionnaire based on the participants’ routine exercise practice and reported a mean increase in score of 1.7 (95% CI: 0.1 to 3.3) from baseline to 12 weeks [27]. A detailed breakdown of the exercise information reported in the included trials is shown in Supplementary Table S1. Individual study related 6MWD and TMWD data are provided in Supplementary Table S2.

### 3.4. Risk of Bias of Included Studies

The risk of bias of the included studies is shown in Table 4. There were some concerns with missing outcomes data in four studies [4,13,15,18]. One study had some concerns in the randomisation process due to baseline testing performed prior to randomisation [29]. One study had some concerns with deviation from the intended intervention [31]. One study had high risk of bias in deviations from the intended interventions and missing outcomes data [33]. One study had a high risk of bias in the selection of reported result. All other domains including randomization process, measurement of outcomes and selection of reported results were at low risk of bias in all other studies. Overall assessment suggested that 12 studies were deemed to be at low risk of bias [4,14,15,16,18,27,28,29,30,31,32,34], and 2 studies were at high risk of bias [13,33].

### 3.5. Network Model

**Figure 2:**
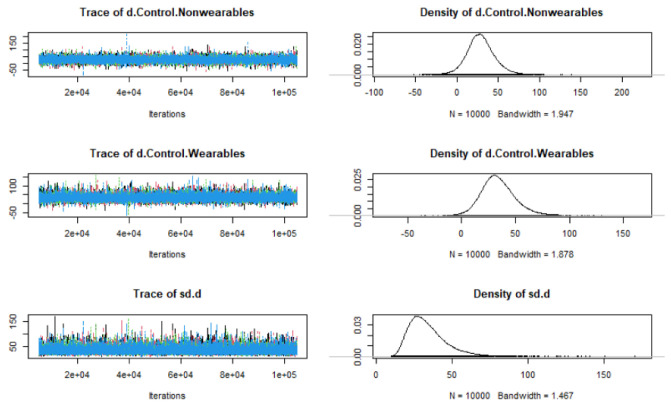
Markov Chain Monte Carlo simulation showing the trace plots and corresponding density plots. Convergence was achieved with higher iterations (100,000) and was suitable for the network model. SD—Standard deviation.

The network model of all treatment arms from all included trials were tested for feasibility of network meta-analysis, and the model convergence was achieved with 100,000 iterations. The Gelman diagnostics showed a PSRF of 1.000445 suggesting the reliability of the network model. The network diagnostics using trace and density plots showed that the models converged and were valid to use (Figure 2).

### 3.6. Effect of the Home Exercise Programs on Walking Distance

**Figure 3:**
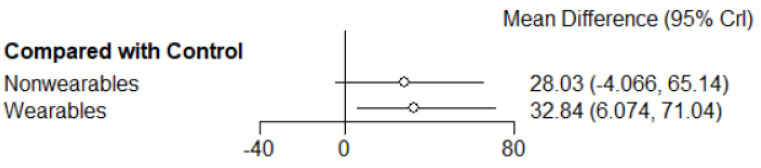
Forest plots of the two intervention strategies suggested a significant improvement of walking distance change score (reported as 6MWD and MWD) in participants who used wearables compared to control group. Results are expressed as mean difference (95% CrI) in metres. Crl: Credible interval. 6MWD—Six-minute walking distance; MWD—Maximum walking distance.

**Figure 4:**
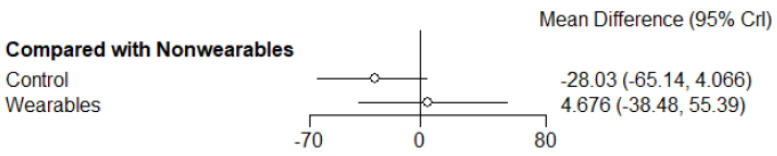
Forest plots suggested no significant improvement of walking distance change score (reported as 6MWD and MWD) in participants who used wearables compared to non-wearables group. Results are expressed as mean difference (95% CrI) in metres. Crl: Credible interval. 6MWD—Six-minute walking distance; MWD—Maximum walking distance.

**Figure 5:**
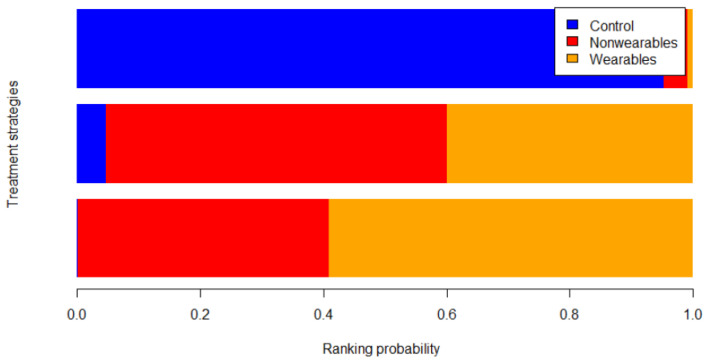
Stacked bar plot showing ranking probability of all treatment strategies. The network model suggests home exercise program informed by wearable activity monitor as the best treatment for improving walking outcomes.

All treatment arms (n = 1526) were connected through 9 arms that tested home exercise programs informed by wearables activity monitors (503 participants), 6 arms that tested home exercise programs not informed by wearable activity monitors (316 participants) and 14 arms with non-exercise control groups (707 participants). The main finding of the NMA was that by comparison to the non-exercise controls, home exercise programs informed by wearable activity monitors significantly improved walking distance (MD: 32.8 m [95% CrI: 6.1, 71.0]). Home exercise programs not informed by wearable activity monitors did not significantly improve walking distance by comparison to non-exercise controls (MD: 28.0 m [95% CrI: −4.1, 65.1]) (Figure 3). There was no significant difference in improvement of walking distance between home exercise programs that were or were not informed by wearable activity monitors (MD: 4.7 m [95% CrI: −38.5, 55.4]) (Figure 4). A SUCRA plot suggested that home exercise programs informed by wearable activity monitor was the best treatment compared to those not informed by wearable activity monitor and non-exercise controls (Figure 5).

Meta-regression of trials testing home exercise programs informed by wearables suggested that presence or absence of telephone counselling (estimate: −37.61; standard error, SE: 24.39; *p* = 0.167), length of the exercise programs (ranging between 6 to 52 weeks) (estimate: −0.47; SE: 0.93; *p* = 0.626) or number of facility visits (ranging between 0 to 26 times) (estimate: −0.28; SE: 2.34; *p* = 0.907) did not significantly influenced walking distance outcomes.

## 4. Discussion

The results of this NMA suggest that home exercise program informed by wearable activity monitors significantly improve walking distance in PAD participants compared to non-exercise controls. The difference of 32.8 m was clinically significant in accordance with the recent study that suggested approximately 8 and 20 metres improvement in 6MWD represented small and large improvement in walking ability, respectively [35]. The improvement in walking distance was not significantly different between home exercise programs informed by as compared with those not informed by wearable activity monitors. There were no trials identified which directly compared the two different types of home exercise programs in the same study. Meta-regression suggested that length of the exercise program, whether or not telephone counselling was provided and number of facility visits did not have an impact on the outcomes.

The benefit of using activity monitors has been examined in populations without PAD. A previous meta-analysis of six RCTs involving 693 participants with coronary heart disease suggested that self-monitoring of physical activity improved daily steps taken by a mean of 2503 (95% CI: 1916, 3090 steps/day, *p* < 0.05) [36]. The largest trial which included 103 participants taking part in cardiac rehabilitation demonstrated that self-monitoring of physical activity using accelerometers significantly increased the mean number of steps per day compared to controls (8609.6 vs. 5512.9 steps, *p* < 0.001) [37]. Another RCT including 64 patients who were recovering from acute coronary syndrome, cardiac surgery or heart failure and who were taking part in cardiac rehabilitation assessed the provision of a step counter (pedometer) for self-monitoring. This increased the overall daily steps walked by a mean (±SD) of 5191 (±3198) steps/day in the first week and by 7890 (±2629) steps/day after one year. A RCT involving 800 healthy participants aged between 21–65 years found that provision of activity monitoring significantly improved moderate-to-vigorous activity (MVPA) at 12 months by 37 MVPA bout min per week (95% CI: 19, 56; *p* = 0.0001) compared to controls. Participants were also categorised into groups receiving incentives with a weekly payment of SGD 15 (Singapore dollar) if they logged between 50,000 and 70,000 steps per week or SGD 30 if they logged 70,000 or more steps per week. When activity monitoring was accompanied by a cash incentive to the participant (mean earning SGD 620) or charity incentive (mean earning SGD 320), the MVPA improved by a mean of 15 MVPA bout min per week (95% CI: –5, 34; *p* = 0.136) and 32 MVPA bout min per week (95% CI: 12, 51; *p* = 0.001), respectively, at 12 months compared to the control group [38].

In addition to improving uptake of exercise, wearable activity monitors may facilitate exercise programs which are more cost-effective than centre-based programs. Centre based supervised exercise therapy has been estimated to cost SGD 14,590 per patient for a program which involved three directly supervised 1-h sessions per week for 26 weeks, supplemented by a subsequent 12-month telephone-based program designed to maintain adherence to exercise [39]. Activity monitors also facilitate tailoring of a home exercise program to individual requirements which is difficult to achieve in group sessions held in centre based supervised exercise therapy [40]. Overall, this past research supports the use of activity monitors to inform home exercise programs but a large head to head trial is needed to definitely test the value of using activity monitoring to inform a home exercise program in patients with PAD.

Seven of the 14 trials reported variable information about adherence to the exercise programs [4,13,14,15,27,29,30]. Only two trials provided the definition for adherence [4,27]. One study reported adherence based on increased number of steps per day by the participants from baseline [29] (Supplementary Table S1). Due to the lack of validated tools for quantitatively measuring adherence, it is not surprising that it was variably reported in the included trials. Agreed criteria for reporting engagement of participants with home exercise programs would be valuable. The methods used to measure exercise intensity varied between a validated seven-item questionnaire in one trial [27] and using the data from activity monitor in five trials [4,13,14,15,30]. Reporting the exercise intensity of the study population is important as a RCT showed that a low-intensity home exercise program did not significantly increase walking distance compared to non-exercise controls [4]. The authors suggested that low-intensity exercise could have encouraged these participants to develop a slower habitual walking pace thus affecting the walking distance at follow-up.

This NMA included largely high quality RCTs with most considered at low risk of bias. A number of limitations of this NMA should be acknowledged. This includes the small number of RCTs identified, the few patients included and the heterogeneous nature of the tested exercise programs. Due to the lack of head-to-head trials available, nodesplit analysis could not be performed to test for inconsistency. Study design and key clinical risk factors, including age, sex, BMI and ABPI, were similar between included trials, suggesting the homogeneity of the included population samples. In addition, follow-up in the included trials varied between 6 and 52 weeks. A previous meta-analysis of RCTs suggested that home exercise programs improved walking distance for duration of less than 12 months but there was no evidence this lasted beyond this point [2]. Therefore, long term trials are needed to investigate whether home exercise programs informed by wearable activity monitors are durable. Importantly, 2 of the included trials reported findings in two publications which were both incorporated in the NMA. While this ensured all outcomes irrespective of time point were included, there was an overlap in the study population. Lastly, no studies required the participants to be familiar with the technology, but training was provided on how to use the activity monitor. However, the authors did not report the digital literacy of participants, which influences the adherence and how generalizable the results of the trial are likely to be. Older age is a key risk factor for PAD and associated with lower rates of digital literacy [41]. Digital literacy likely influenced the uptake of activity monitoring informed home exercise program. Future studies are needed to examine methods to achieve broad uptake in the older population with PAD.

In conclusion, this study suggests that home exercise programs informed by wearable activity monitors improve walking distance in patients with PAD. It is unclear, however, whether exercise programs using activity monitoring are more effective than programs not using activity monitoring.

## Figures and Tables

**Table 1:** Baseline characteristics of participants in the included studies.

Reference	Group	Sample Size	Age (Years)	Male Gender (%)	ABPI	BMI	Currently Smoking (%)	Diabetes (%)	CHD (%)	MI (%)	HTN (%)	Dylipidemia (%)	Stroke (%)	Classic Claudication Symptoms (%)	Exertion Leg Pain Other than Claudication (%)	No Exertional Leg Pains (%)	History of Leg Revascularisation (%)
McDermott 2021 [4]	Intervention	124	68.8 (8.7)	51.6	0.67 (0.15)	31.1 (7.3)	23.4, 57.3 *	42.7	NR	25.8	89.5	NR	NR	NR	NR	NR	NR
	Control	65	69.5 (10.1)	50.8	0.67 (0.15)	30.8 (7.3)	21.5, 58.3 *	53.9	NR	10.8	80.0	NR	NR	NR	NR	NR	NR
McDermott 2018 [15]	Intervention	99	70.1 (10.6)	46.0	0.65 (0.15)	29.6 (5.3)	79.8 ^	35.4	NR	16.2	NR	NR	NR	17.2	68.7	14.1	36.4
	Control	101	70.4 (10.1)	49.0	0.67 (0.14)	29.9 (5.3)	90.1 ^	31.7	NR	20.8	NR	NR	NR	21.8	66.3	11.9	43.6
Tew 2015 [16]	Intervention	14	69.1 (7.6)	71.4	0.67 (0.17)	27.9 (3.5)	71.0 *	7.0	14.0	NR	64.0	NR	0.0	NR	NR	NR	NR
	Control	9	67.8 (14.1)	66.7	0.64 (0.18)	29.6 (7.4)	56.0 *	22.0	33.0	NR	78.0	NR	11.0	NR	NR	NR	NR
Gardner 2014 [13]	Intervention	53	67.0 (10.0)	52.0	0.68 (0.24)	29.0 (5.7)	35.0	40.0	35.0	17.0	88.0	93.0	25.0	NR	NR	NR	37.0
	Control	51	65.0 (9.0)	60.0	0.74 (0.21)	29.0 (6.1)	42.0	37.0	28.0	18.0	83.0	87.0	10.0	NR	NR	NR	27.0
McDermott 2013 [14]	Intervention	88	69.3 (9.5)	50.5	0.67 (0.16)	29.1 (7.0)	26.8	28.9	NR	13.4	NR	NR	9.3	32.0	24.7	8.3	NR
	Control	90	71.0 (9.6)	49.5	0.67 (0.18)	29.0 (6.5)	22.7	37.1	NR	14.4	NR	NR	15.5	23.7	29.9	8.3	NR
McDermott 2014 [18]	Intervention	81	69.9 (9.2)	48.2	0.67 (0.16)	28.7 (6.5)	24.7	30.9	NR	13.6	NR	NR	8.6	70.4	NR	NR	NR
	Control	87	72.0 (9.3)	49.4	0.68 (0.18)	29.0 (6.7)	18.4	36.8	NR	13.8	NR	NR	17.2	75.9	NR	NR	NR
Bearne 2022 [27]	Intervention	95	67.6 (8.7)	69.0	0.63 (0.12)	26.7 (5.7)	86.0 ^	36.0	NR	NR	59.0	NR	NR	89.0	44.0	0.0	30.0
	Control	95	68.2 (9.0)	71.0	0.63 (0.12)	26.9 (5.8)	90.0 ^	32.0	NR	NR	63.0	NR	NR	94.0	60.0	0.0	25.0
Duscha 2018 [29]	Intervention	10	66.1 (9.8)	80	0.6 ± 0.2	26.6 ± 4.5	100.0	10.0	60.0	NR	60.0	100	NR	NR	NR	NR	NR
	Control	9	73.1 (4.7)	88.9	0.6 ± 0.1	31.2 ± 7.6	100.0	33.3	66.7	NR	77.8	88.9	NR	NR	NR	NR	NR
Gardner 2011 [30]	Intervention	29	65.0 (11.0)	45	0.72 (0.23)	29.9 (5.6)	10.0	43.0	NR	NR	88.0	90.0	NR	NR	NR	NR	NR
	Control	30	65.0 (10.0)	54	0.76(0.22)	29.7(6.9)	10.0	31.0	NR	NR	79.0	85.0	NR	NR	NR	NR	NR
Brenner 2020 [33]	Intervention	18	68.6 (6.9)	67	NR	27.6 (5.2)	44.0	NR	NR	NR	NR	NR	NR	NR	NR	NR	NR
	Control	15	63.7 (8.5)	60	NR	26.4 (5.2)	53.0	NR	NR	NR	NR	NR	NR	NR	NR	NR	NR
Collins 2011 [28]	Intervention	72	66.2 (10.2)	75	0.96 (0.4)	35.0 (9.3)	10.0	NR	NR	NR	62.0	54.0	NR	NR	NR	NR	NR
	Control	73	66.8 (10.1)	80	0.94 (0.5)	33.7 (7.0)	18.0	NR	NR	NR	57.0	54.0	NR	NR	NR	NR	NR
Sandercock 2007 [34]	Intervention	15	62.0 (14.0)	80	0.60 (0.10)	27.1 ± 4.2	40.0	NR	NR	NR	NR	NR	NR	NR	NR	NR	NR
	Control	15	67.0 (6.0)	66.7	0.60 (0.10)	27.7 ± 6.7	46.7	NR	NR	NR	NR	NR	NR	NR	NR	NR	NR
Collins 2019 [32]	Intervention	57	62.9 (9.7)	82.5	0.86 (0.14)	31.6 (7.9)	66.7 ^	36.8	NR	NR	NR	NR	5.3	NR	NR	NR	NR
	Intervention	57	65.9 (11.1)	22.8	0.87 (0.14)	32.8 (16.3)	57.9 ^	31.6	NR	NR	NR	NR	3.5	NR	NR	NR	NR
	Control	60	63.9 (12.5)	26.7	0.84 (0.15)	34.4 (10.0)	58.3 ^	38.3	NR	NR	NR	NR	1.7	NR	NR	NR	NR
Larsen and Lassen 1966 [31]	Intervention	7	58 (7)	86.0	NR	NR	NR	NR	NR	NR	NR	NR	NR	NR	NR	NR	NR
	Control	7	56 (6)	100.0	NR	NR	NR	NR	NR	NR	NR	NR	NR	NR	NR	NR	NR
**Summary statistics**	**Intervention**	**819**	**66.4 (0.3)**	**61.8**	**0.71 (0.01)**	**29.5 (0.3)**	**48.2**	**31.1**	**36.3**	**17.2**	**72.9**	**84.3**	**8.6**	**52.2**	**45.8**	**7.5**	**34.5**
	**Control**	**707**	**67.1 (0.4)**	**62.3**	**0.70 (0.01)**	**29.8 (0.3)**	**48.2**	**35.3**	**42.6**	**15.6**	**74.0**	**78.7**	**11.1**	**53.9**	**52.1**	**6.7**	**31.9**

Age, ABPI and BMI are presented as Mean (SD). ABPI—Ankle brachial pressure index; BMI—Body mass index; CHD—Coronary heart disease; HTN—Hypertension; MI—Myocardial infarction; NR—Not reported. * Ex-smoker; ^ either current or ex-smoker. For the summary statistics, ex-smoker data were used when current smoking data were not reported.

**Table 2:** Structure of the tested exercise program.

Reference	Program Duration (Weeks)	Number of Facility Visits	Sessions per Week	Duration of Sessions	Face to Face Meeting	Educational Counselling	Online Counselling	Telephone Counselling	Behavioural Counselling	Adherence (%)
McDermott 2021 [4]	52	4	5 times per week	50 min	Yes	Yes	No	Yes	Yes	85.1 ⸷ 91.9 ⸶
McDermott 2018 [15]	36	4	Variable, but typically 5 days per week	10–15 min working up to 50 min	Yes	Yes	No	Yes	Yes	73.9 ⸷ 92.0 ⸶
Tew 2015 [16]	6	6	NR	30 min and increase daily total steps to more than 7500	Yes	Yes	No	Yes	Yes	NR
Gardner 2014 [13]	12	4	3 days per week at a self-selected pace	Progressively increased from 20 to 45 min per session	Yes	No	No	No	No	80.6 ⸸ 81.0 δ
McDermott 2013 [14]	24	24	5 times per week	Working up to 50 min per session	Yes	Yes	No	No	Yes	84.0 #
McDermott 2014	52	26	At least 5 days per week at home	up to 50 min	Yes	Yes	No	Yes	Yes	NR
Bearne 2022 [27]	12	2	3 times per week	At least 30 min of walking per day	Yes	Yes	No	Yes	Yes	exercise adherence rating scale 1.7 (95% CI: 0.1 to 3.3)
Collins 2019 [32]	52	26	3–5 times per week	30 to 50 min	Yes	Yes	No	Yes	Yes	NR
Duscha 2018 [29]	12	0	NR	NR	No	Yes	Yes	Yes	No	NR *
Gardner 2011 [30]	12	6	3 times per week	20 min for the first 2 weeks; then progressive increase by 5 min biweekly until total of 45 min walking achieved	Yes	No	No	No	No	NR *
Brenner 2020 [33]	12	1	5 times per week	Until minimal claudication pain	No	No	No	Yes	No	No
Collins 2011 [28]	24	24	4 times per week	50 min and increase step count by 50 each session	Yes	Yes	No	Yes	Yes	No
Sandercock 2007 [34]	12	0	3 times per week	30 min	No	No	No	Yes	No	No
Larsen and Lassen 1966 [31]	24	9	Once daily	60 min including rest time	Yes	No	No	No	No	No

NR—Not reported. * Adherence was discussed but no data for specific groups were provided. ⸶ Onsite visits. ⸷ Scheduled intervention calls. ⸸ Exercise sessions completed. δ Overall. # Attendance of Group-Mediated Cognitive Behavioural Intervention.

**Table 3:** Use of the activity data for counselling.

Reference	Name of Wearable	Location of Wearable	Frequency of Wearing	Data Viewable to the Patient	Data Used by Patient for Self-Motivation	Data Used by Investigators for Counselling	Frequency and Format of Counselling
McDermott 2021 [4]	Accelerometer	Worn on the hip	Participants wore their accelerometer during each session and uploaded accelerometer data on exercise frequency, time, and intensity onto the study website using a home computer or tablet provided by the study.	Yes	Yes	Yes	Accelerometer data were viewable to a coach who telephoned participants weekly for 12 months and helped them adhere to their prescribed exercise.
McDermott 2018 [15]	Accelerometer (Actigraph), FitBit Zip, FitBit Inc.)	Worn on the right hip NR	Accelerometer was worn all the time on the right hip and removed only for bathing or sleeping	Yes	Yes	Yes	Feedback from patients was used to design an appealing home-based exercise intervention. Telephone counselling was provided monthly.
Tew 2015 [16]	Accelerometer (ActiGraph GT3X+, ActiGraph, Pensacola, FL)	NR	Participants were encouraged to wear their pedometer on a daily basis and to self-monitor their ambulatory activity and intensity of claudication during each session using a specifically-designed exercise diary.	Yes	Yes	Yes	Participants were supported in developing short and long term goals for walking, with reference to their baseline daily steps count recorded by wearing an accelerometer for seven days before attending the workshop. They were also supported in developing an action plan detailing where, when and how their first initial goal will be reached and they are encouraged to repeat this process for each new goal. Two weeks after the educational workshop, participants were contacted by telephone to review progress and discuss goal setting and barriers
	Pedometer (Yamax SW-200 Digi-Walker)	NR					
Gardner 2014 [13]	Step-activity monitor (StepWatch3TM, Orthoinnovations, Inc., Oklahoma City, OK)	Right ankle	Patients wore the step activity monitor during each session, and returned the monitor and a logbook to the research staff at the end of week 1, 4, 8, and 12.	Yes	Yes	Yes	During each visit, patients had a brief 15-min meetings, monitor data were downloaded, results were reviewed, and feedback was provided for the upcoming month of training.
Bearne 2022 [27]	Pedometer (Yamax Digi-Walker SW-200)	NR	Participants recorded where, when, and with whom they would walk and established ways to self-monitor their walking exercise.	Yes	Yes	Yes	Walking exercise goals and plans were agreed upon collaboratively with the physical therapist and included identifying progressive, individualized walking targets. Participants received an intervention manual that included an exercise diary, with goal setting, problem-solving, and action planning worksheets.
Duscha 2018 [29]	Fitbit Charge device (Fitbit, Inc., San Francisco, CA)	Wrist	Wore the device for 2 weeks continuously during waking hours	Yes	No	Yes	Participants were provided a personalized exercise prescription based on steps per day. Study staff had access to patient on-line accounts so that they could better support technical problems, monitor physical activity, and provide motivation and feedback during the study.
Gardner 2011 [30]	Step activity monitor (StepWatch 3™)	Right ankle	Worn during each session and exercise log book	Yes	Yes	Yes	15-min meetings with exercise physiologist at 1, 2, 4, 6, 8, 10 and 12 weeks to discuss activity based on step monitor and exercise log, and to give new instructions on exercise duration.
Collins 2011 [28]	Pedometer	NR	Worn during each exercise session	No	No	Yes	Counselling based on PACE at entry and through biweekly phone calls; entry exercise training (2 sessions) and weekly group walking sessions with an instructor
Larsen and Lassen 1966 [31]	Pedometer	NR	Participants were given pedometer with instructions to take a daily walk besides their normal physical activity.	Yes	No	Yes	Advised to record pedometer step count after each walk; reviewed (once per week in month 1 and monthly for months 2–6) to discuss step count and encouraged to continue

NA—Not applicable; NR—Not reported; PAD—Peripheral artery disease.

**Table 4:** Risk of bias assessed using Revised Cochrane risk-of-bias tool for randomized trials (RoB 2).

Reference	Randomisation Process	Deviations from the Intended Interventions	Missing Outcome Data	Measurement of Outcomes	Selection of the Reported Result	Overall Quality Assessment
McDermott 2021 [4]	(+)	(+)	(±)	(+)	(+)	Low
McDermott 2018 [15]	(+)	(+)	(±)	(+)	(+)	Low
Tew 2015 [16]	(+)	(+)	(+)	(+)	(+)	Low
Gardner 2014 [13]	(+)	(+)	(±)	(+)	(−)	High
McDermott 2013 [14]	(+)	(+)	(+)	(+)	(+)	Low
McDermott 2014 [18]	(+)	(+)	(±)	(+)	(+)	Low
Bearne 2022 [27]	(+)	(+)	(+)	(+)	(+)	Low
Duscha 2018 [29]	(±)	(+)	(+)	(+)	(+)	Low
Gardner 2011 [30]	(+)	(+)	(+)	(+)	(+)	Low
Brenner 2020 [33]	(+)	(−)	(−)	(+)	(+)	High
Collins 2011 [28]	(+)	(+)	(+)	(+)	(+)	Low
Sandercock 2007 [34]	(+)	(+)	(+)	(+)	(+)	Low
Collins 2019 [32]	(+)	(+)	(+)	(+)	(+)	Low
Larsen and Lassen 1966 [31]	(+)	(±)	(+)	(+)	(+)	Low

(+) Low risk of bias; (±) Some concerns; (−) High risk of bias.

## Data Availability

Data is contained within the article or Supplementary Materials.

## Supplementary Materials

The following supporting information can be downloaded at: https://www.mdpi.com/article/10.3390/s22208070/s1, Table S1: Definition and components of adherence from the reported trials; Table S2: Mean change in outcomes in each group from included studies.
