# Supplementary material for: Network Meta-Analysis of Trials Testing If Home Exercise Programs Informed by Wearables Measuring Activity Improve Peripheral Artery Disease Related Walking Impairment
Source: Sensors (Basel). 2022 Oct 21;22(20):8070. doi: 10.3390/s22208070 (PMC9611238; doi:10.3390/s22208070)
Supplement: Supplementary file 1 [file sensors-22-08070-s001.zip › sensors-1949301-supplementary.pdf]

sensors

MDPI

Supplementary material

Search terms:

((Peripheral arterial disease[Title/Abstract] OR Peripheral artery disease[Title/Abstract] OR peripheral vascular disease[Title/Abstract] OR PAD[Title/Abstract] OR lower extremity arterial disease[Title/Abstract]) AND ((((((Walking) OR (6MWT)) OR (6MWD)) OR (Treadmill)) OR (Claudication)) OR (MWD))) AND (Exercise[Title/Abstract])

Supplementary Table S1. Definition and components of adherence from the reported trials.

Reference	Adherence definition	Exercise adherence reporting (patient reported/objective measurement)	Number of exercise sessions completed per week	Percentage of exercise sessions completed (Yes/No), %	How exercise intensity/effort level was measured	Exercise intensity/effort level at longest follow up time point	Attained exercise target intensity (Yes/No), %	Attended all scheduled onsite visits (Yes/No), %	Attended all scheduled telephone calls (Yes/No), %
Bearne 2022 [1]	(Score range, 0 to 24 [24 indicates best]; minimal clinically important difference, 5.5 points.	Objectively measured using a 6-item questionnaire of EARS.	NR	NR	Participants reported energy expenditure (MET min/week) using a 7-item questionnaire	3846 ± 6192 MET min/week	NR	81.2	74.4
McDermott 2021 [2]	>80% of the participant's individualized goal for the number of minutes	Patient reported	3.5 ± 1.5 [range, 0.1-6.4 days]	NR	Individualised intensity accelerometer counts	(median intensity, 1584 [range, 229-4710] activity units)	63.0	91.9	85.1

	exercised per week								
McDermott 2018 [3]	NR	Patient reported	3.5 ± 4.2	NR	Physical activity outcome using accelerometer counts	111,767 ± 72,263	NR	93.0	79.2
Gardner 2011 [4]	NR	NR	NR	82.5±27.7	By converting cadence of each home-based exercise session to an average speed of walking, and then estimating oxygen uptake from walking speed	2832 ± 1491 MET min/week	NR	NR	NR
Gardner 2014 [5]	NR	NR	NR	80.6±27.4		2472 ± 1564 MET min/week	NR	NR	NR
McDermott 2013 [6]	NR	NR	NR	NR	Using a vertical accelerometer	866.1 ± 405.4 activity units	NR	84.0	NR
Duscha 2018 [7]	NR	NR	NR	NR	NR	NR	NR	NR	NR

MET was measured using international physical activity questionnaire. EARS – Exercise Adherence Rating Scale. MET – Metabolic equivalents. NR – Not reported.

Supplementary Table S2. Mean change in outcomes in each group from included studies.

Study name	Group	Outcome	Mean change (m)	SD
Bearne 2022 [1]	Wearables	6MWD	22.3	1.3
Bearne 2022 [1]	Control	6MWD	9.2	1.4
McDermott 2021 [2]	Wearables	6MWD	33.1	70.7
McDermott 2021 [2]	Control	6MWD	-10.6	60.5
McDermott 2018 [3]	Wearables	6MWD	2.9	69.5
McDermott 2018 [3]	Control	6MWD	12.0	61.6
Tew 2015 [8]	Wearables	6MWD	22.9	59.5
Tew 2015 [8]	Control	6MWD	-20.7	50.9
Gardner 2014 [5]	Wearables	6MWD	44.0	72.5
Gardner 2014 [5]	Control	6MWD	4.0	49.3
McDermott 2013 [6]	Non-wearables	6MWD	42.4	63.1
McDermott 2013 [6]	Control	6MWD	-11.1	66.6
McDermott 2014 [9]	Non-wearables	6MWD	26.5	65.3
McDermott 2014 [9]	Control	6MWD	-7.0	62.8
Collins 2019a [10]	Non-wearables	6MWD	13.75	6.1
Collins 2019a [10]	Control	6MWD	-1.08	5.7
Collins 2019b [10]	Non-wearables	6MWD	-7.75	5.6
Duscha 2018 [7]	Wearables	TMWD	202.0	255.0
Duscha 2018 [7]	Control	TMWD	20.0	96.0
Gardner-2011 [4]	Wearables	TMWD	110.0	172.0
Gardner-2011 [4]	Control	TMWD	-9.0	156.0
Brenner 2020 [11]	Non-wearables	TMWD	124.0	125.8
Brenner 2020 [11]	Control	TMWD	26.0	120.6
Collins 2011 [12]	Wearables	TMWD	24.0	166.0
Collins 2011 [12]	Control	TMWD	39.0	167.0
Sandercock 2007 [13]	Non-wearables	TMWD	59.0	138.0
Sandercock 2007 [13]	Control	TMWD	37	191.0
Larsen 1966 [14]	Wearables	TMWD	402	391.0
Larsen 1966 [14]	Control	TMWD	-14	82.0

6MWD – Six minute walking distance, m – Meters, TMWD – Treadmill maximum walking distance, SD – Standard deviation.

References

1. Bearne, L.M., et al., *Effect of a Home-Based, Walking Exercise Behavior Change Intervention vs Usual Care on Walking in Adults With Peripheral Artery Disease: The MOSAIC Randomized Clinical Trial.* JAMA, 2022. **327**(14): p. 1344-1355.
2. McDermott, M.M., et al., *Effect of Low-Intensity vs High-Intensity Home-Based Walking Exercise on Walk Distance in Patients With Peripheral Artery Disease: The LITE Randomized Clinical Trial.* JAMA, 2021. **325**(13): p. 1266-1276.
3. McDermott, M.M., et al., *Effect of a Home-Based Exercise Intervention of Wearable Technology and Telephone Coaching on Walking Performance in Peripheral Artery Disease: The HONOR Randomized Clinical Trial.* JAMA, 2018. **319**(16): p. 1665-1676.
4. Gardner, A.W., et al., *Efficacy of quantified home-based exercise and supervised exercise in patients with intermittent claudication: a randomized controlled trial.* Circulation, 2011. **123**(5): p. 491-8.
5. Gardner, A.W., et al., *Step-monitored home exercise improves ambulation, vascular function, and inflammation in symptomatic patients with peripheral artery disease: a randomized controlled trial.* J Am Heart Assoc, 2014. **3**(5): p. e001107.
6. McDermott, M.M., et al., *Home-based walking exercise intervention in peripheral artery disease: a randomized clinical trial.* JAMA, 2013. **310**(1): p. 57-65.
7. Duscha, B.D., et al., *Effects of a 12-Week mHealth Program on FunctionalCapacity and Physical Activity in Patients With PeripheralArtery Disease.* Am J Cardiol, 2018. **122**(5): p. 879-884.
8. Tew, G.A., et al., *The development and pilot randomised controlled trial of a group education programme for promoting walking in people with intermittent claudication.* Vasc Med, 2015. **20**(4): p. 348-57.
9. McDermott, M.M., et al., *Home-based walking exercise in peripheral artery disease: 12-month follow-up of the GOALS randomized trial.* J Am Heart Assoc, 2014. **3**(3): p. e000711.
10. Collins, T.C., et al., *Efficacy of Community-Based Exercise Therapy Among African American Patients With Peripheral Artery Disease: A Randomized Clinical Trial.* JAMA Netw Open, 2019. **2**(2): p. e187959.
11. Brenner, I.K.M., et al., *Low-Intensity Exercise Training Increases Heart Rate Variability in Patients With Peripheral Artery Disease.* Biol Res Nurs, 2020. **22**(1): p. 24-33.
12. Collins, T.C., et al., *Effects of a home-based walking intervention on mobility and quality of life in people with diabetes and peripheral arterial disease: a randomized controlled trial.* Diabetes Care, 2011. **34**(10): p. 2174-9.
13. Sandercock, G.R., et al., *The impact of short term supervised and home-based walking programmes on heart rate variability in patients with peripheral arterial disease.* J Sports Sci Med, 2007. **6**(4): p. 471-6.
14. Larsen, A. and N.A. Lassen, *EFFECT OF DAILY MUSCULAR EXERCISE IN PATIENTS WITH INTERMITTENT CLAUDICATION.* The Lancet, 1966. **288**(7473): p. 1093-1095.
